# Supplementary figures and images for: In silico identification of natural product inhibitors against Octamer-binding transcription factor 4 (Oct4) to impede the mechanism of glioma stem cells
Source: PLoS One. 2021 Oct 6;16(10):e0255803. doi: 10.1371/journal.pone.0255803 (PMC8494328; doi:10.1371/journal.pone.0255803)

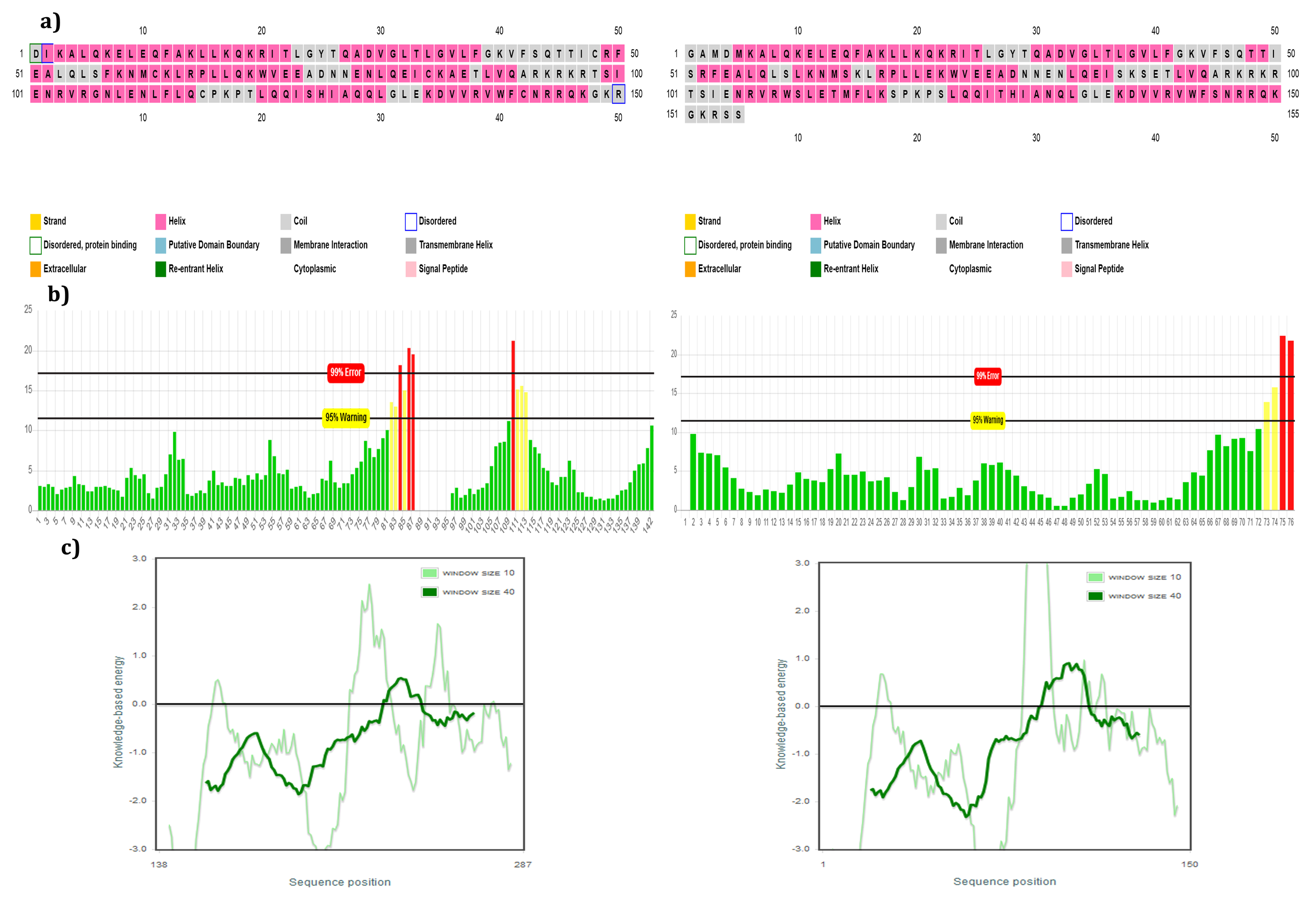

Supplement: S1 Fig — a) Secondary structure; b) ERRAT analysis; c) ProSA knowledge based energy analysis of both model structure and template structure. (TIF) [file pone.0255803.s001.tif]

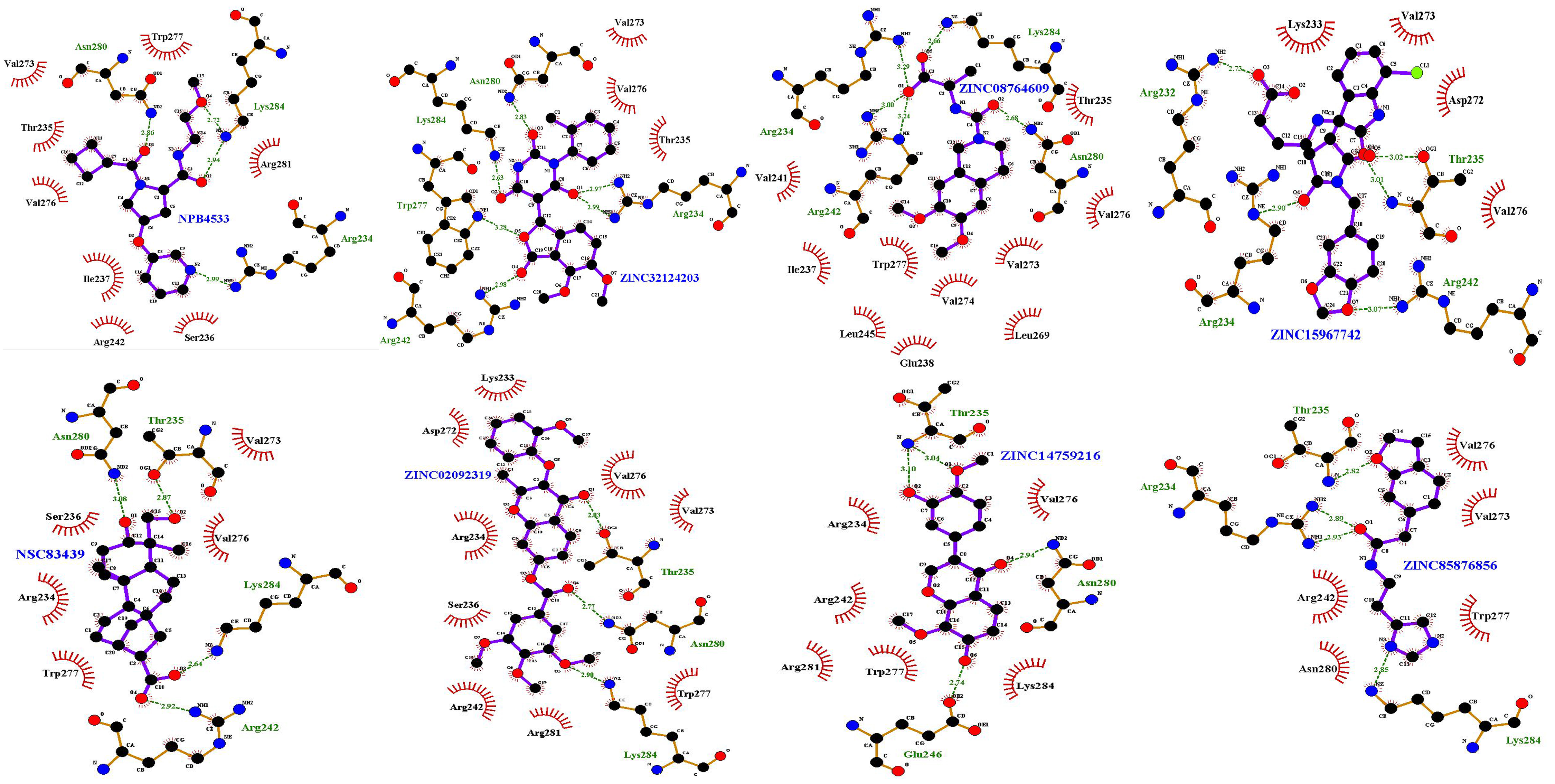

Supplement: S2 Fig — (TIF) [file pone.0255803.s002.tif]

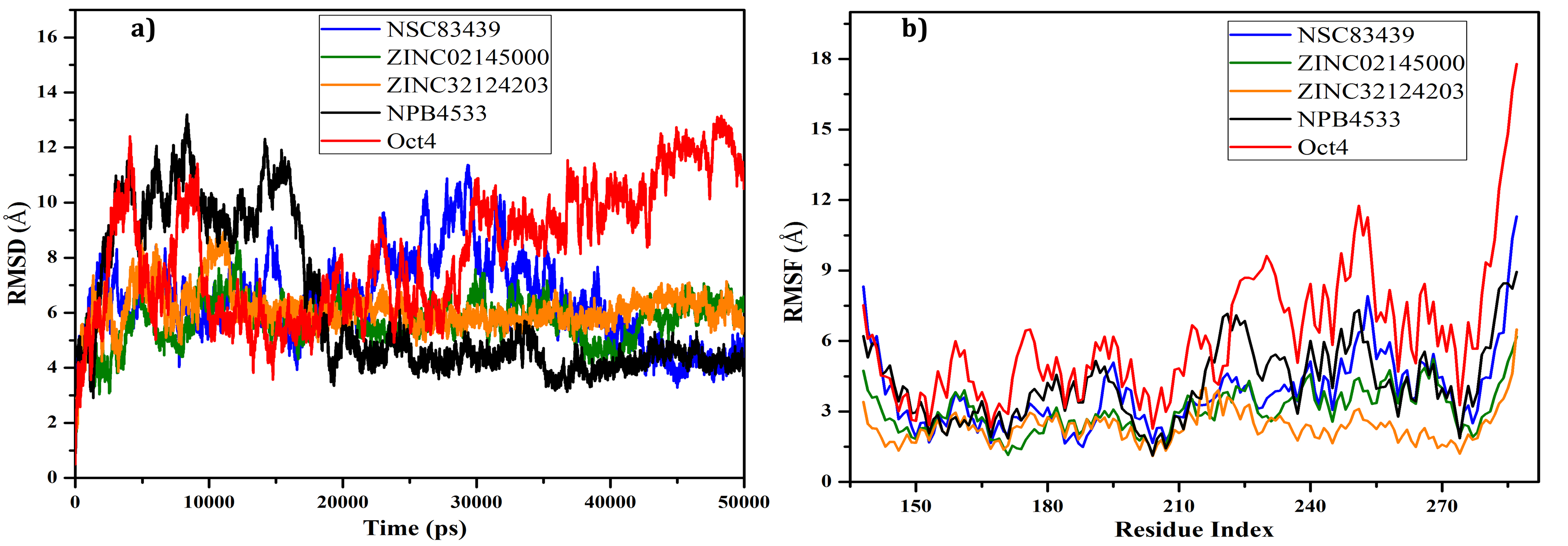

Supplement: S3 Fig — a) Represents backbone RMSD of complexes relative to the starting complexes; b) Displays RMSF plot of C-alpha fluctuations of selected complexes. (TIF) [file pone.0255803.s003.tif]

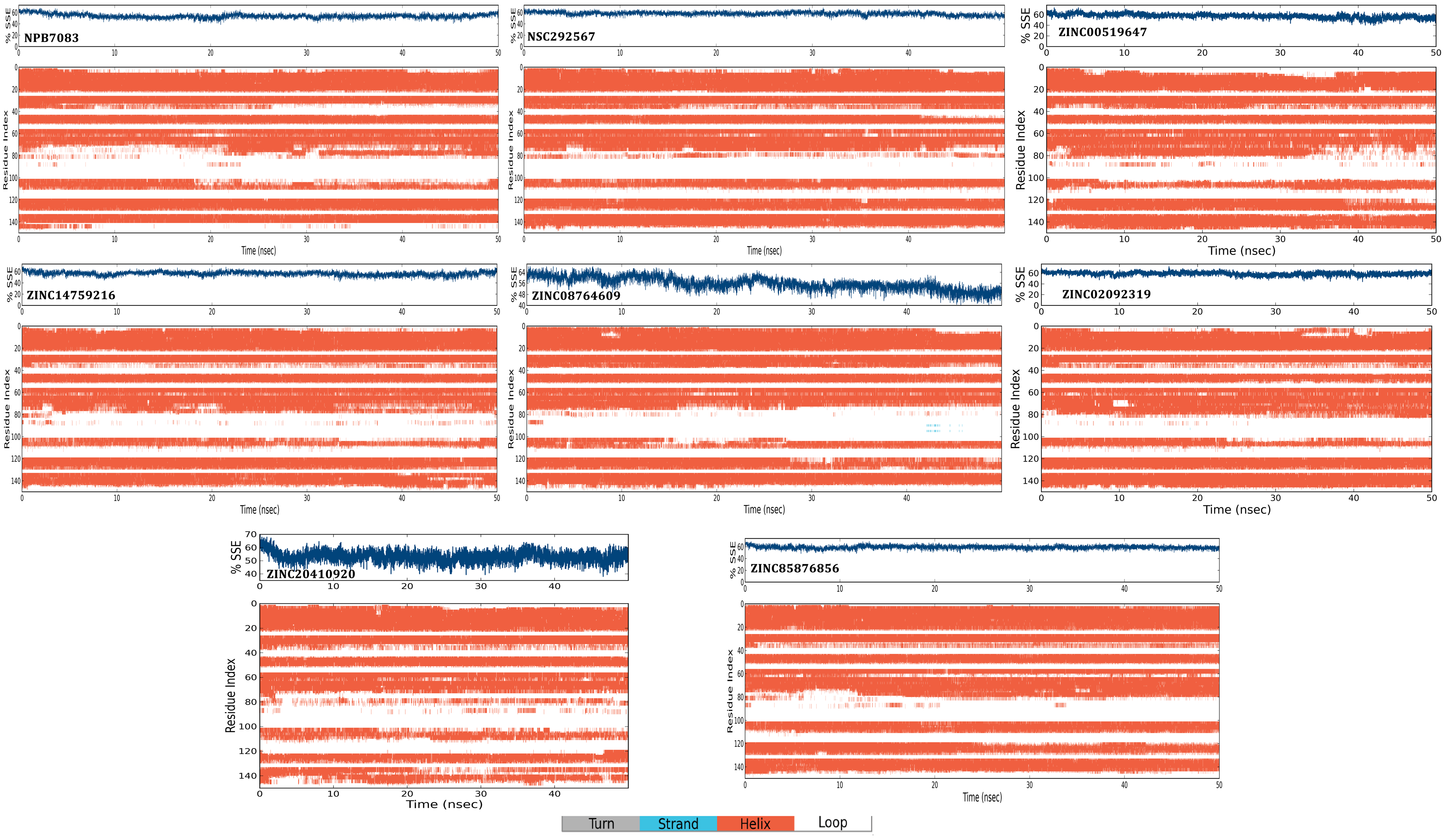

Supplement: S4 Fig — (TIF) [file pone.0255803.s004.tif]

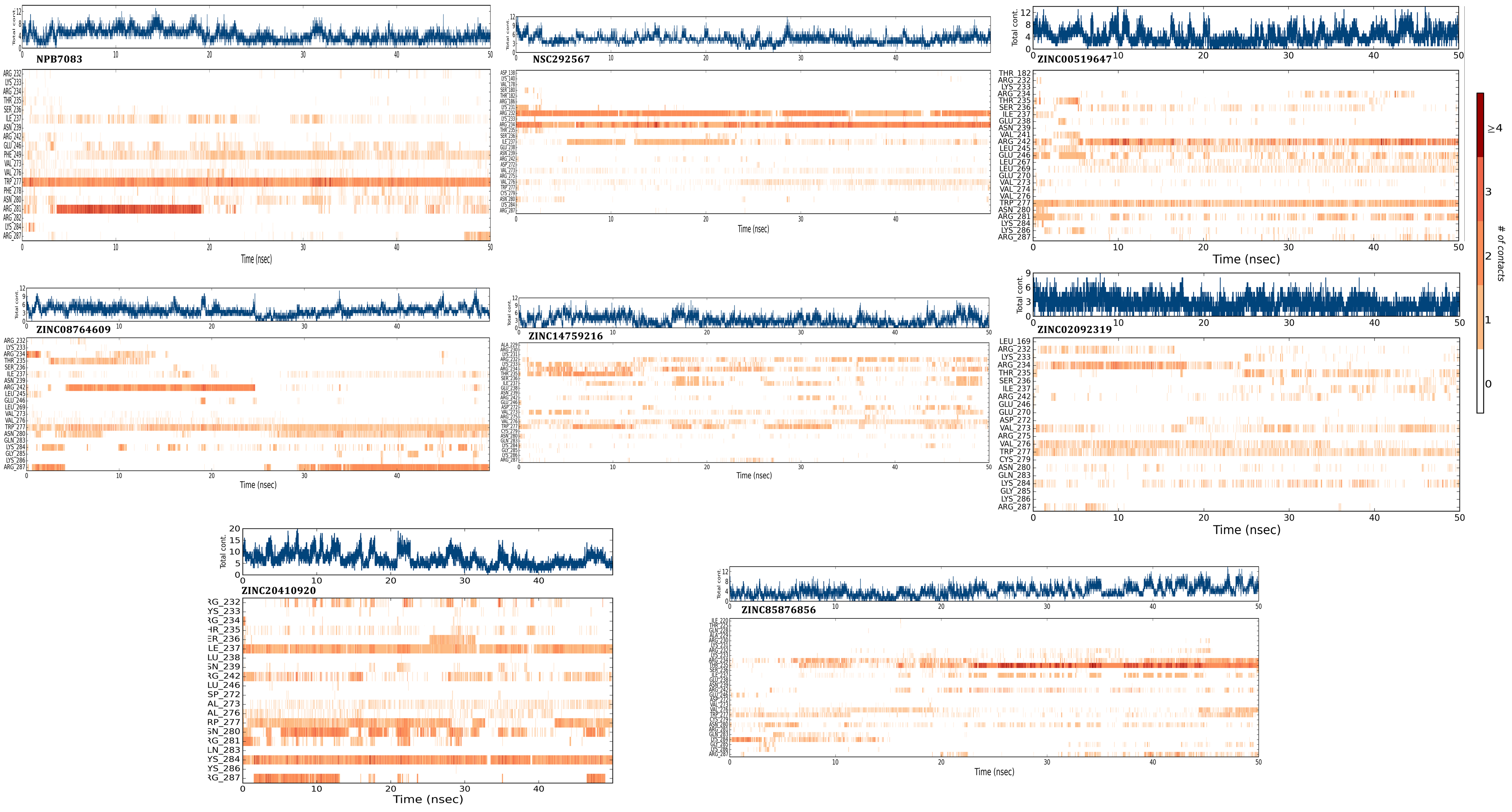

Supplement: S5 Fig — (TIF) [file pone.0255803.s005.tif]
