# Supplementary material for: In silico identification of natural product inhibitors against Octamer-binding transcription factor 4 (Oct4) to impede the mechanism of glioma stem cells
Source: PLoS One. 2021 Oct 6;16(10):e0255803. doi: 10.1371/journal.pone.0255803 (PMC8494328; doi:10.1371/journal.pone.0255803)
Supplement: S1 File — (DOCX) [file pone.0255803.s006.docx]

**SUPPORTING INFORMATION**

**In silico identification of natural product inhibitors against Octamer-binding transcription factor 4 (Oct4) to impede the mechanism of glioma stem cells**

Chirasmita Nayak and Sanjeev Kumar Singh*

**Affiliation**

Computer Aided Drug Design and Molecular Modeling Lab, Department of Bioinformatics,

Alagappa University, Karaikudi-630004, Tamil Nadu, India.

***Corresponding Author**

**Dr. Sanjeev Kumar Singh**

Email: skysanjeev@gmail.com

Tel: +91-9894429800

Fax : +91 4565 225202

**Supplementary Tables:**

**S1 Table:** XP docking results representing docking energy and interacting amino acid residues

| **Sl No.** | **Compound ID** | **Docking score** | **Glide energy** | **Glide e-model** | **Interacting amino acid residues** |
| --- | --- | --- | --- | --- | --- |
|  | NPB7083 | -5.411 | -42.044 | -55.399 | N280, K284, T235, R234 |
|  | NPB4569 | -5.066 | -45.104 | -60.486 | N280, K284 |
|  | NPB4533 | -4.968 | -41.269 | -44.685 | R234, T235, K284, N280 |
|  | NPB2106 | -4.868 | -37.382 | -50.726 | R234, T235, N280 |
|  | NPB4579 | -4.710 | -37.335 | -51.500 | K284, R234, T235 |
|  | NPB4526 | -4.696 | -40.937 | -56158 | T235, R234, K284 |
|  | NPB1717 | -4.598 | -33.431 | -39.996 | K284, T235, N280 |
|  | NSC292567 | -7.670 | -41.933 | -43.945 | T235, R234,R232 |
|  | NSC83439 | -6.842 | -24.246 | -28.919 | T235, R232, N280 |
|  | NSC83436 | -5.751 | -23.230 | -32.691 | R234, R232 |
|  | NSC607097 | -5.502 | -31.125 | -34.125 | R234, K284, W277 |
|  | ZINC00489055 | -6.047 | -29.071 | -35.132 | N280, K284 |
|  | ZINC00519647 | -6.041 | -33.057 | -41.998 | K284, N280, T235, R234 |
|  | ZINC02092319 | -5.927 | -48.372 | -60.094 | K284, N280, W277, T235 |
|  | ZINC02145000 | -6.713 | -35.703 | -39.919 | R234, W277,N280 |
|  | ZINC04026084 | -6.324 | -27.235 | -32.627 | N280, T235, K231 |
|  | ZINC04073984 | -6.168 | -26.477 | -32.823 | R232, K231, N280 |
|  | ZINC04084707 | -6.391 | -20.945 | -25.669 | N280, T235 |
|  | ZINC04090192 | -6.399 | -31.500 | -44.211 | K284, T235, T277 |
|  | ZINC08764609 | -5.946 | -37.995 | -46.628 | T235, K284, N280, W277 |
|  | ZINC14410961 | -6.076 | -26.665 | -31.751 | T235, K233, R232 |
|  | ZINC14759216 | -5.894 | -36.530 | -44.152 | K284, N280, T235, W277 |
|  | ZINC15967741 | -6.410 | -42.699 | -51.822 | T235, N280,R232 |
|  | ZINC15967742 | -7.386 | -42.421 | -53.834 | T235, N280, R232 |
|  | ZINC17744426 | -6.633 | -21.739 | -28.205 | K284, N280 |
|  | ZINC20410920 | -6.381 | -47.737 | -56.502 | N280, K284, R234 |
|  | ZINC32124203 | -5.933 | -38.414 | -49.371 | K284, N280, T235, R234 |
|  | ZINC35455596 | -6.328 | -27.357 | -34.676 | R232, N280 |
|  | ZINC35457235 | -6.490 | -30.032 | -45.240 | T235, N280 |
|  | ZINC85876856 | -5.942 | -37.268 | -52.158 | K284, N280, W277, T235 |

**S2 Table:** Predicted ADME properties of selected thirteen compounds

| **Sl. No** | **Compound ID** | **mol_MW** | **CNS** | **donorHB** | **accptHB** | **QPlogPo/w** | **QPlogS** | **QPlogBB** | **%Human-Oral Absorption** |
| --- | --- | --- | --- | --- | --- | --- | --- | --- | --- |
|  | NPB4533 | 347.41 | -1 | 1.00 | 9.45 | 0.99 | -2.64 | -0.72 | 85.10 |
|  | NPB 7083 | 579.64 | -1 | 3.00 | 10.40 | 4.27 | -5.11 | -0.99 | 91.80 |
|  | NSC 83439 | 334.45 | -1 | 1.00 | 4.70 | 3.12 | -3.97 | -0.96 | 77.93 |
|  | NSC 292567 | 724.97 | -1 | 3.00 | 12.55 | 6.62 | -6.81 | -0.68 | 73.42 |
|  | ZINC00519647 | 334.28 | -1 | 2.00 | 8.00 | 0.683 | -2.36 | -0.90 | 74.96 |
|  | ZINC02092319 | 492.48 | -1 | 0.00 | 8.50 | 4.45 | -5.87 | -0.76 | 100.00 |
|  | ZINC02145000 | 364.37 | -1 | 2.00 | 7.50 | 1.86 | -3.44 | -0.74 | 87.17 |
|  | ZINC08764609 | 308.33 | -1 | 1.25 | 4.75 | 2.39 | -3.43 | -0.87 | 77.12 |
|  | ZINC14759216 | 314.29 | -1 | 2.00 | 5.50 | 2.07 | -3.44 | -0.99 | 87.32 |
|  | ZINC15967742 | 497.89 | -1 | 2.00 | 9.00 | 0.77 | -4.20 | -0.93 | 49.14 |
|  | ZINC20410920 | 444.35 | -1 | 5.00 | 9.75 | 0.46 | -2.34 | -0.95 | 42.57 |
|  | ZINC32124203 | 410.38 | -1 | 2.00 | 8.00 | 2.09 | -3.43 | -0.71 | 87.90 |
|  | ZINC85876856 | 271.31 | 0 | 2.00 | 5.25 | 1.72 | -2.97 | -0.67 | 89.17 |

| Molecular weight of the molecule (**mol_MW**) | 130.0 – 725.0 |
| --- | --- |
| Predicted central nervous system activity on a –2 (inactive) to +2 (active) scale (**CNS**) | –1 to +2 |
| Hydrogen bond donor (**donorHB**) | 0.0 – 6.0 |
| Hydrogen bond accepter (**accptHB**) | 2.0 – 20.0 |
| Predicted octanol/water partition coefficient (**QPlogPo/w**) | –2.0 – 6.5 |
| Predicted aqueous solubility (**QPlogS**) | –6.5 – 0.5 |
| Predicted brain/blood partition coefficient (**QPlogBB**) | –3.0 – 1.2 |
| Predicted human oral absorption on 0 to 100% scale (**PercentHuman-OralAbsorption**) | <25% is poor  >80% is high |
